# Secondary hypogonadism following hand, foot, and mouth disease in an adult: a case report and review of literature

**DOI:** 10.1186/s12879-022-07030-0

**Published:** 2022-01-15

**Authors:** Zhaoying Chen, Chen Jiang, Xiaoyu Cheng, Lidan Ma, Ying Xin, Tian Liu, Ruixia Sun

**Affiliations:** 1 Department of Endocrinology and Metabolism, The Affiliated Hospital of Qingdao University, Qingdao, 266003 China; 2 Department of Gastroenterology, The Affiliated Hospital of Qingdao University, Qingdao, 266003 China

**Keywords:** Hand-foot-mouth disease, Hypogonadism, Adult-onset, Case report

## Abstract

**Background:**

Previous reports have described hypogonadism associated with virus infection such as hantavirus, human immunodeficiency virus (HIV) or severe acute respiratory syndrome coronavirus 2 (SARS-COV-2). However, to our best knowledge there has been no case report of secondary hypogonadism following hand, foot, and mouth disease (HFMD).

**Case presentation:**

A previously healthy 28-year-old man with no history of major physical and psychological trauma, presented with bilateral gynecomastia and erectile dysfunction 2 weeks after HFMD. Laboratory testament showed the level of gonadotropin hormones declined. Imaging examination demonstrated no major abnormal change in pituitary or reproductive system. The diagnosis of hypogonadism was established. Then the patient was ordered to maintain mental health outward of hospital without drug intervention. One month after presentation, his gonadotropin hormone level and sexual desire had recovered, while bilateral gynecomastia and erectile dysfunction symptoms disappeared.

**Conclusions:**

Physicians should notice the possibility for hypogonadism in adult patients with a recent history of HFMD.

**Supplementary Information:**

The online version contains supplementary material available at 10.1186/s12879-022-07030-0.

## Background

Hand, foot, and mouth disease (HFMD) is a common disease caused by different types of viruses [1]. Most serotypes of enterovirus A group, such as coxsackievirus (CV) A2, A4, A5, A8, A10, A16 and enterovirus type 71 (EV71), and some serotypes of enterovirus B group, such as CVB2, B4, and few echovirus (ECV) are related to its pathogenesis [2]. Adult HFMD may be caused by more virulent viruses, particularly CVA6. Usually, HFMD occurs in children under 5 years of age and present with a mild and self-limited syndrome [3, 4]. However, adults, especially those living with infected children, may suffer from HFMD. Complications of HFMD involve nail shedding, pulmonary edema, myocardial impairment, purpura and neurological sequelae [3, 5, 6]. Hypogonadism following HFMD in sexual mature adult has not been reported before. Here, we present the first case report of secondary hypogonadism associated with HFMD.

## Case presentation

A previously healthy 28-year-old male presented with bilateral gynecomastia and erectile dysfunction for 1 month. The man with normal growth and development was sexually active before, denying history of major physical and psychological trauma. He also had no contact history with chemical, radioactive or toxic substances. The patient had seen a doctor at local hospital for the symptoms of fever, herpes on hands, feet as well as mouth and was diagnosed as HFMD. He recovered quickly after using ibuprofen and ribavirin for 2 days. 2 weeks after the diagnosis, the patient developed the symptoms of hyposexuality, erectile dysfunction with bilateral breast pain and development and clear nipple discharge. He came to our endocrinologist office, and the physical exam showed the patient had gynaecomastia at P2 according to the Tanner-Stage score system, and a ductile mass with a diameter of about 2 cm can be palpated under the nipple, which secreted clear discharge after squeezing. Testicular volume was evaluated about 15 ml, pubic hair P5, with penis length 12 cm, circumference 9 cm. His olfactory function was normal. Laboratory findings showed the level of gonadotropin and gonadal hormones declined. Luteinizing hormone (LH) level was 1.15 mIU/ml (normal range 1.70–8.60 mIU/ml), follicle-stimulating hormone (FSH) level was 1.26 mIU/ml (normal range 1.50–12.40 mIU/ml), testosterone (T) level was 3.19 nmol/l (normal range 9.90–27.80 mIU/ml), estradiol (E_2_) level was below 18.35 pmol/l (normal range 99.40–192.00 mIU/ml). Progesterone (P, normal range < 0.159–0.474 nmol/l), insulin (INS, normal range 2.6–24.9 μIU/ml), growth hormone (GH, normal range 0.03–2.47 ng/ml), insulin-like growth factor-1 (IGF-1, normal range 60-350 ng/ml), thyroid stimulating hormone (TSH, normal range 0.51–4.30 μIU/ml), free triiodothyronine (T3, normal range 3.10–6.80 pmol/l), free thyroxine (T4, normal range 12.6–21.0 pmol/l) were within normal range. In addition, the result of overnight low-dose dexamethasone suppression test was normal (Cortisol, COR < 50 nmol/L). There was no evident abnormity index showing in carbohydrate antigen 19–9 (CA19-9), carbohydrate antigen 125 (CA125), carcino-embryonic antigen (CEA), alpha-fetoprotein (AFP) and human chorionic gonadotropin-β (HCG-β) (Additional file 1: Table S1). Semen examination revealed lower sperm count, density and motility. The patient was subjected to a GnRH intramuscular injection (i.m.) infusion test (triptorelin, 100 μg). Our result (Additional file 1: Table S2) showed that peak LH level (more than 3 times compared to the baseline) after an i.m. infusion test of GnRH occurred at 60 min, while FSH had no noticeable change. With respect to imaging examinations, breast ultrasound image demonstrated gland-like echo at both sides (1.7 × 1.5 × 0.7 cm on the right side and 1.6 × 1.6 × 0.8 cm on the left side). The male reproductive system ultrasound showed calcification of prostate and cyst of right caput epididymidis. Plain chest computerized tomography (CT) showed multiple ground glass attenuation in both lungs and suspicious infectious lesions. Plain adrenal CT and Dynamic enhanced magnetic resonance imaging (MRI) showed there were no obvious abnormal changes in pituitary or adrenal.

Following these conditions, the diagnosis of hypogonadism was established. Clinicians speculated that the disease was caused by HFMD-associated virus attacking hypothalamic neurons. The patient received no drug intervention and was ordered to keep a good mood outward of hospital. The function of hypothalamic-pituitary–gonadal axis (HPG axis) should be revalued after a 1-month observation period when medications are not recommended. The patient came back for a return visit after the 1-month follow-up. The recovery of gonadotropin hormone level was shown in Additional file 1: Table S3. Other laboratory findings were negative. Semen examination was normal. The patient reported morning erection, spermatorrhea, recovery of sexual desire and satisfying sex life of 4–5 times per week. Physical examination found that his bilateral breast mass and nipple discharge had disappeared.

## Discussion and conclusion

To the best of our knowledge this is the first report of HFMD-related hypogonadism in an adult. HFMD is an infectious disease caused by Enteroviruses (EVs). Usually, HFMD affects children younger than 5 years old, however it can still affect adults [3, 7]. HFMD patients could present with neurological injury and complications, according to previous researches, the main neurological manifestations are myoclonic jerks, aseptic meningitis or encephalitis [8, 9]. So, this patient’s clinical course is quite unique for second hypogonadism in adult occurring following HFMD. Laboratory tests of the case showed evidently reduced levels of gonadotropins and sex hormones while other anterior pituitary hormones showed no deficiency, which suggested HFMD-induced damage of central nervous system could lead to hypothalamic and/or pituitary dysfunction.

Some previous reports have described secondary hypogonadism associated with HIV, hantavirus and SARS-CoV-2 virus infection [10–13], and several mechanisms of such severe complication were presented. For HIV infection, an observational cohort study (CHAMPS) conducted in New York found that among HIV-infected males, uncontrolled HIV viraemia (910,000 copies/ml) was significantly associated with secondary hypogonadism [14]. The cause of hypogonadism in HIV-infected men could be multi-factorial related to damage of either hypothalamic or pituitary areas by tumor or inflammation and specific drugs use such as exogenous androgen. For hantavirus infection, previous studies have found that hantavirus infection can cause 56% patients presenting with low serum gonadal hormone levels [11], the mechanism involved could be virus-induced central nervous system infection leading to transient or permanent hypothalamic and/or pituitary dysfunction [15]. There may also be immune factors, including excessive production of pro-inflammatory factors in acute phase of infection, leading to immune-mediated endothelial cell injury [16]. In the case of COVID-19, a previous research conducted on 81 male patients of COVID-19, which reported lower serum testosterone levels, higher LH levels [17]. Other studies implied SAR-CoV2 infection might lead to hypogonadism via multiple possible mechanisms such as testicular injury, oxidative stress and psychological stress [10, 18].

Secondary hypogonadism following HFMD may also share the mechanisms with hypogonadism caused by the other virus infection. In this case, the patient experienced complete pubertal development, thus congenital factors could be excluded. Psychological factors might not be the initial factor of sexual dysfunction since patient denied the history of psychological trauma and had no emotional discord with his sexual partner. Also, the patient had no history of consuming drugs acting on endocrine system and no evidence of the vascular abnormalities, peripheral neuropathy and testicular disease like orchitis was found. Besides, the result of GnRH stimulating test showed that the pituitary function was well-behaved and there was no pituitary adenoma, empty sella or other positive manifestations according to pituitary dynamic enhanced MRI. Therefore, the mechanism involved HFMD-associated hypogonadism seems to be that infection induce hypothalamic and/or pituitary dysfunction and suppress activity of hypothalamic–pituitary–testicular axis, causing reduced luteinizing hormone (LH) and testosterone levels.

In summary, HFMD virus infection could be an etiologic factor of hypogonadism. Physicians should notice the possibility for hypogonadotropic hypogonadism in adult patients with a recent history of HFMD.

## Supplementary Information

**Additional file 1: Table S1.** Laboratory Examination Data on Admission. **Table S2. **Data of GnRH agonist stimulating test. **Table S3.** Endocrinological Examination Data after HFMD.

## Data Availability

All relevant data to this case are included within the article.

## Abbreviations

HFMD: Hand, foot, and mouth disease
CT: Computed tomography
GnRH: Gonadotropin-releasing hormone
