# Supplementary material for: Secondary hypogonadism following hand, foot, and mouth disease in an adult: a case report and review of literature
Source: BMC Infect Dis. 2022 Jan 15;22:56. doi: 10.1186/s12879-022-07030-0 (PMC8760742; doi:10.1186/s12879-022-07030-0)
Supplement: Supplementary file 1 — Additional file 1: Table S1. Laboratory Examination Data on Admission. Table S2. Data of GnRH agonist stimulating test. Table S3. Endocrinological Examination Data after HFMD. [file 12879_2022_7030_MOESM1_ESM.docx]

**Supplement Table 1. Laboratory Examination Data on Admission**

| **Complete Blood Count** | **Biochemistry** | **Endocrinological Examination** | **Others** |
| --- | --- | --- | --- |
| WBC 5,830/μl | ALT 10IU/l | GH 0.1ng/ml | CEA 1.66ng/ml |
| RBC 440×10⁴ /μl | AST 14IU/l | IGF-1 72.53ng/ml | CA19-9 26.94U/ml |
| Hb 13.5g/dl | TG 1.16mmol/l | INS 5.31uIU/ml | CA125 7.45U/ml |
|  | TC 3.28mmol/l | COR 423.00nmol/l | AFP 0.64ng/ml |
|  | HDL-c 1.76mmol/l | PTH 25.84pg/ml |  |
|  | LDL-c 1.13mmol/l  Cr 57umol/l  BUN 5.87mmol/l | LH 1.15mIU/ml  FSH 1.26mIU/ml |  |
|  | Glu 4.57mmol/l | β-HCG <0.10IU/l |  |
|  | K 4.35mmol/l | PL 354.30mIU/l |  |
|  | Na 143.90mmol/l | T 3.19nmol/l |  |
|  | Ca 2.16mmol/l | E₂ <18.35pmol/l |  |
|  | P 0.83mmol/l | P <0.16nmol/l |  |
|  | Mg 0.89mmol/l |  |  |

**Supplement Table 2. GnRH agonist stimulating test**

|  | 0min | 30min | 60min | 90min | 120min |
| --- | --- | --- | --- | --- | --- |
| LH(mIU/ml) | 1.15 | 1.71 | 64.11 | 60.67 | 56.46 |
| FSH(mIU/ml) | 0.67 | 1.26 | 0.63 | 2.40 | 2.61 |

100μg triptorelin was given i.m. s.t.

**Supplement Table 3. Endocrinological Examination Data after HFMD**

|  | 1m after | 2m after |
| --- | --- | --- |
| LH | 1.15mIU/ml | 8.83mIU/ml |
| FSH | 1.26mIU/ml | 2.36mIU/ml |
| PL | 354.3mIU/l | 160.1mIU/l |
| T | 3.19nmol/l | 36.60nmol/l |
| E₂ | <18.35pmol/l | 99.56pmol/l |
